# Supplementary material for: Role of B Cell Lymphoma 2 in the Regulation of Liver Fibrosis in miR-122 Knockout Mice
Source: Biology (Basel). 2020 Jul 8;9(7):157. doi: 10.3390/biology9070157 (PMC7408427; doi:10.3390/biology9070157)

Raw data for fibrosis paper

WT and LKO liver lysates to check BCL2 (Fig. 3g)

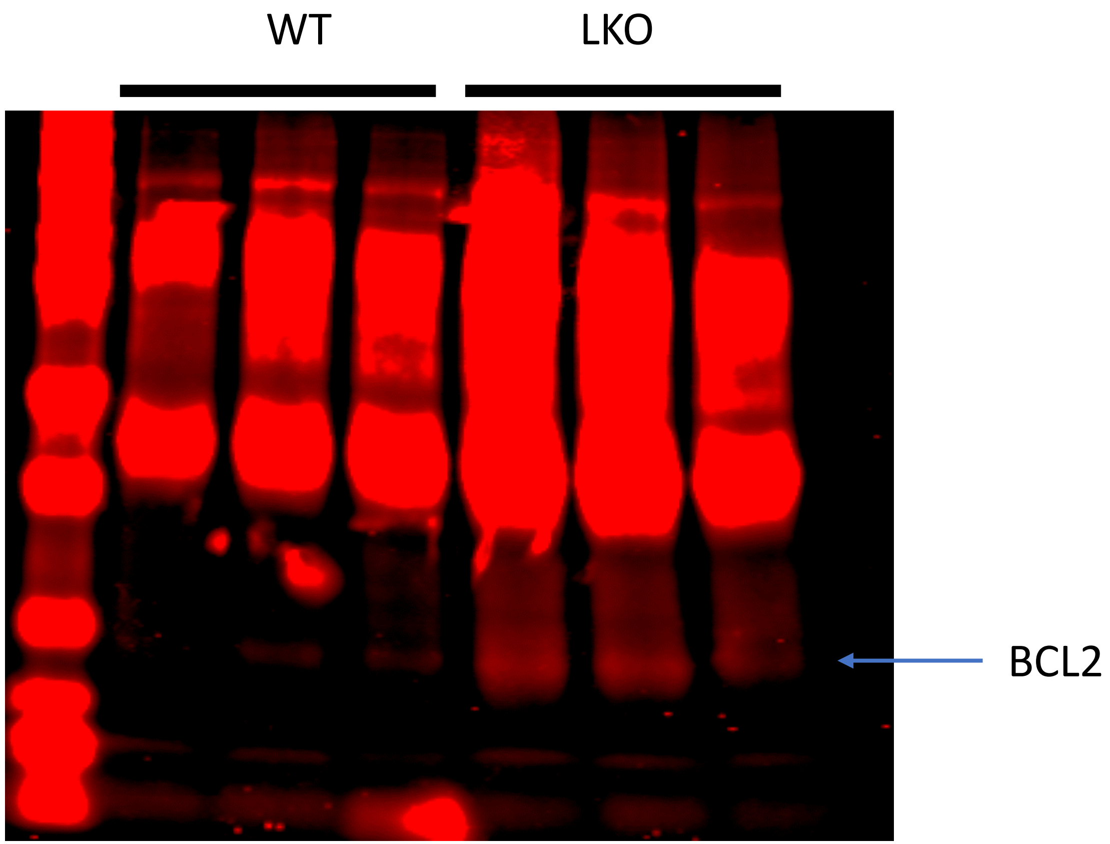

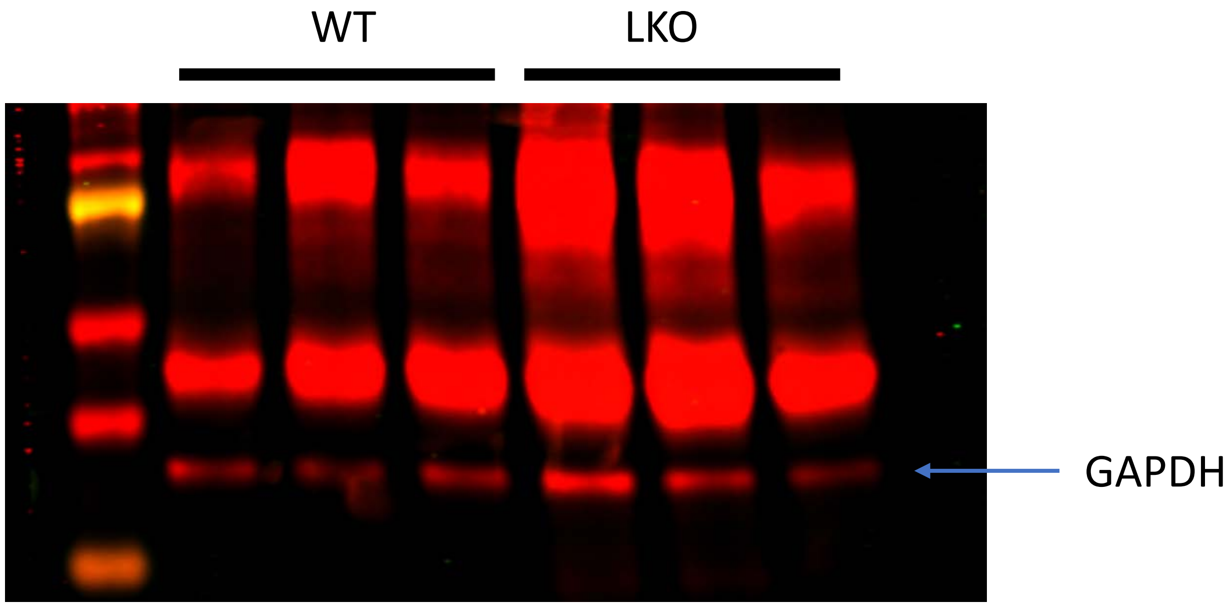

siRNA of c-myc in LX2 cells (72hr) (Fig.4E)

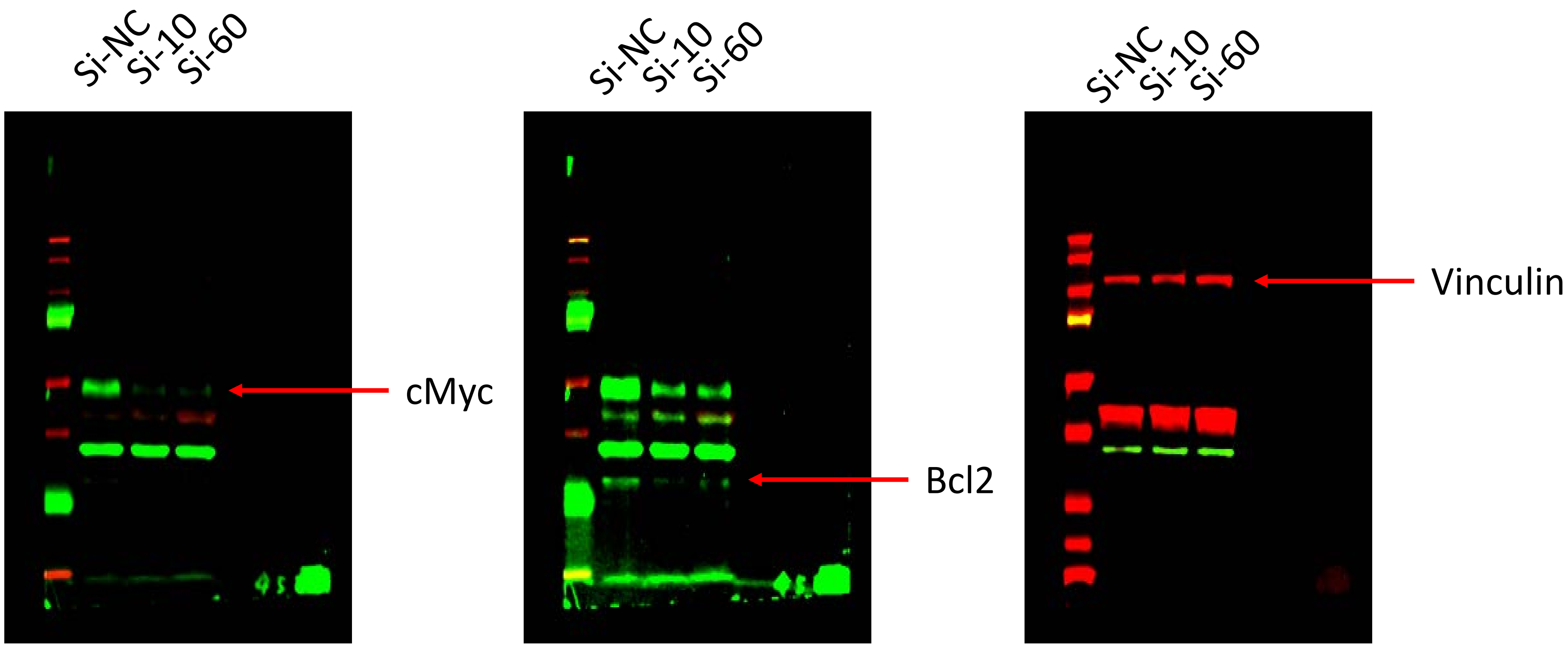

KY cell line (LX-2) treat with DMSO and VEN 10uM (Fig. s8)

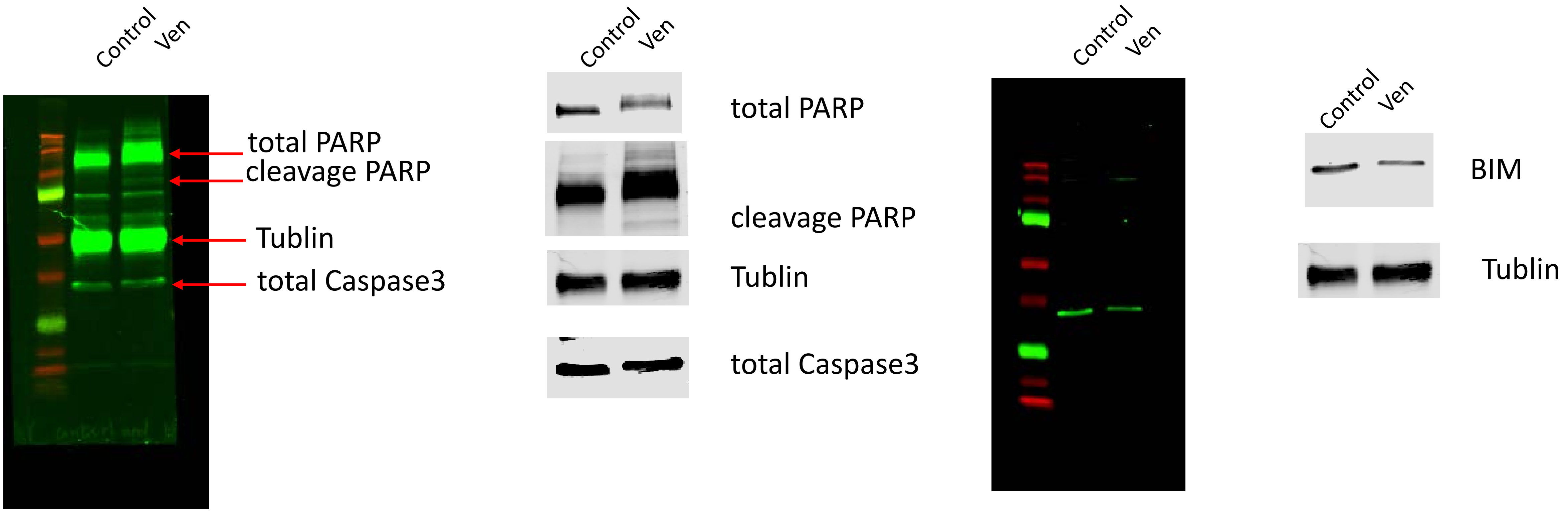

EC4 cell line treated with Doxycycline to check myc and BCL2 (Fig. S6)

These two samples were running with Juan's samples (labeled as "JB sample")

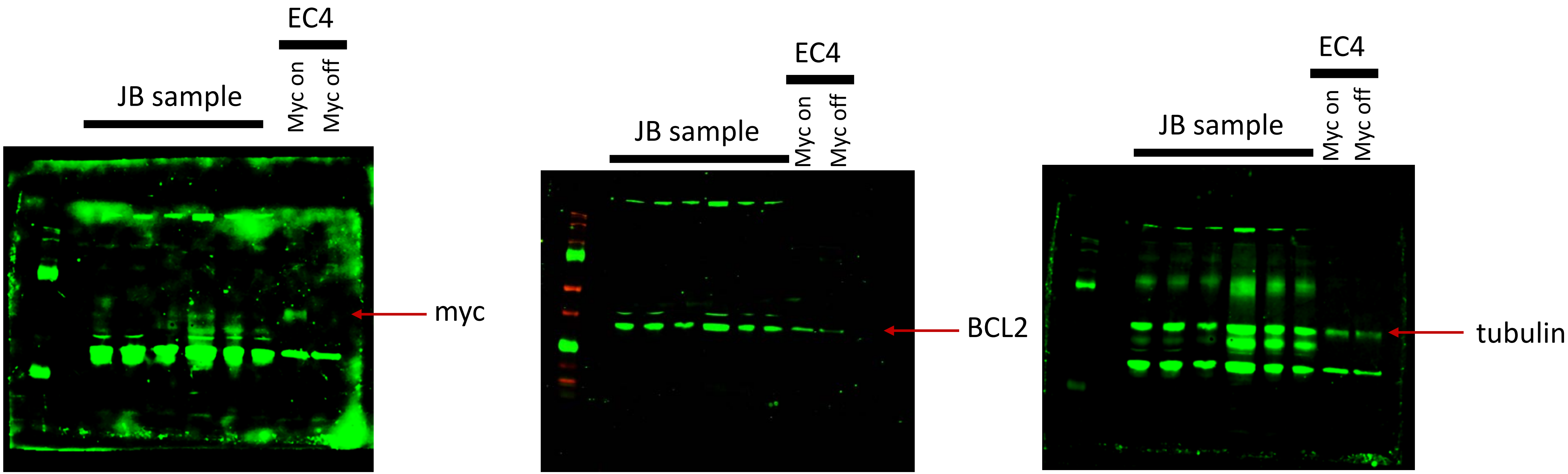

Supplement: Supplementary file 1 [file biology-09-00157-s001.zip › biology-826085-non-published.pdf]
