# Supplementary material for: Role of B Cell Lymphoma 2 in the Regulation of Liver Fibrosis in miR-122 Knockout Mice
Source: Biology (Basel). 2020 Jul 8;9(7):157. doi: 10.3390/biology9070157 (PMC7408427; doi:10.3390/biology9070157)
Supplement: Supplementary file 1 [file biology-09-00157-s001.zip › biology-826085-supplementary.pdf]

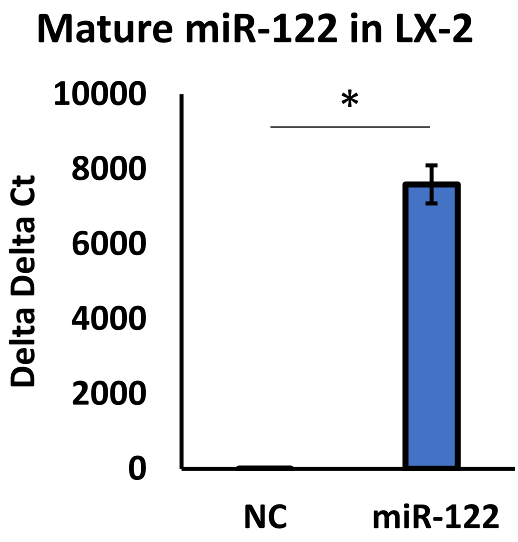

Supplementary Figure 1. miR-122 expression in transfected LX-2 cells.

(A) Mature miR-122 level was evaluated using Taqman RT-qPCR assay in the LX-2 cells transfected with 25nM of scramble RNA or miR-122 mimic RNA using Taqman RT-qPCR assay for 48 hours. *P<0.05, Student's t-test.

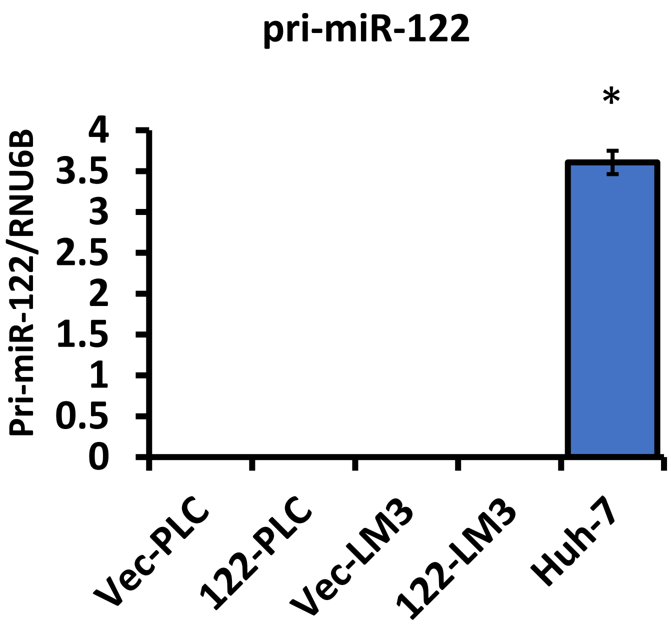

Supplementary Figure 2. Detection of primary transcripts of miR-122 (pri-miR-122).

pri-miR-122 was not detectable (no CT values) in LX-2 cells HCC co-cultured with HCC cells in transwell chambers. Huh7 was a positive control for the Taqman RT-qPCR assay of pri-miR-122. *P<0.05, Student's t-test.

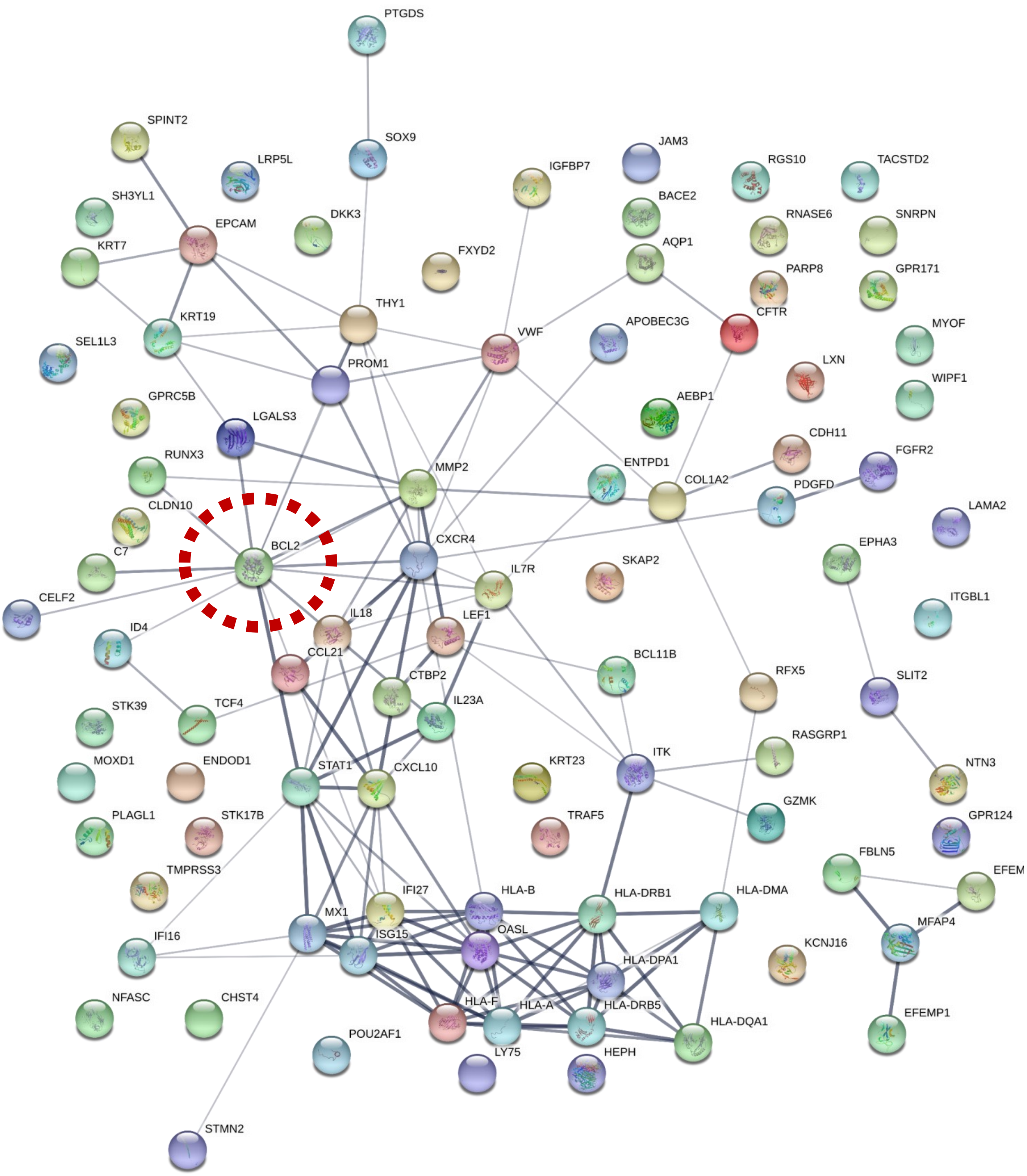

Supplementary Figure 3. Top 100 upregulated genes in Cirrhotic livers. Gene list were retrieved from Oncomine data base (https://www.oncomine.org). BCL2 (circled red) is one of the hub genes as demonstrated by functional protein association networks: STRING (https://string-db.org) with the interaction score set to 0.4 (Version 10.5).

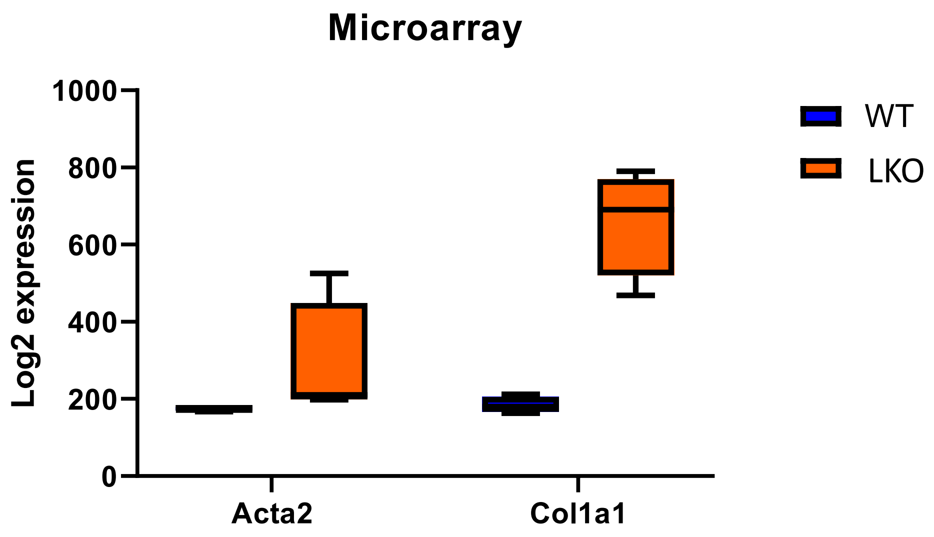

Supplementary Figure 4. *Acta2* and *Col1a1* gene expression levels determined from the microarray analysis of the WT and miR-122 LKO livers. Microarray data retrieved from the wildtype (WT, n=4) and miR-122 LKO (n=4) livers. Data presented as min to max and median is indicated. *P<0.05, Student's t-test.

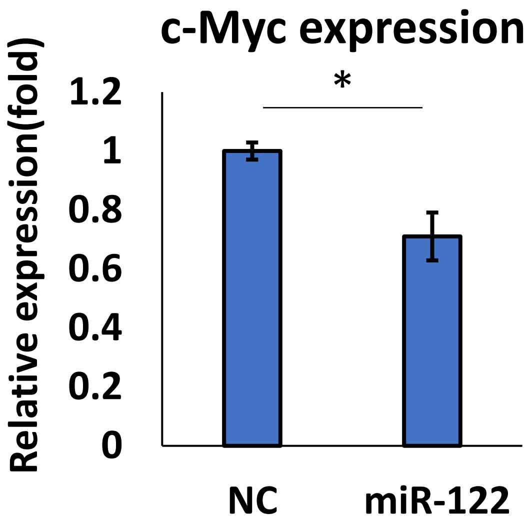

Supplementary Figure 5. c-MYC expression in the miR-122 transfected LX-2 cells. c-MYC expression was evaluated in the LX-2 cells transfected with scramble RNA or miR-122 mimic (25nM) by RT-qPCR assay at 48 hours. *P<0.05, Student's t-test.

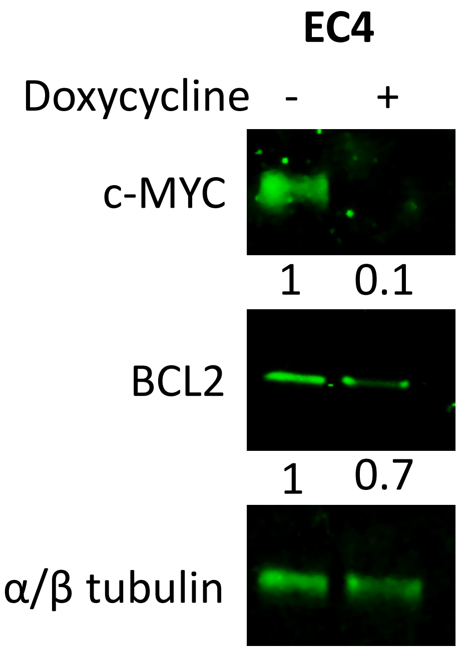

Supplementary Figure 6. BCL2 protein level is regulated by c-MYC. BCL2 protein levels in EC4 cells, a c-MYC driven, tumor-derived mouse HCC cell line. c-MYC level could be repressed by culturing in presence of doxycycline 20ng/ml for 24 hour.

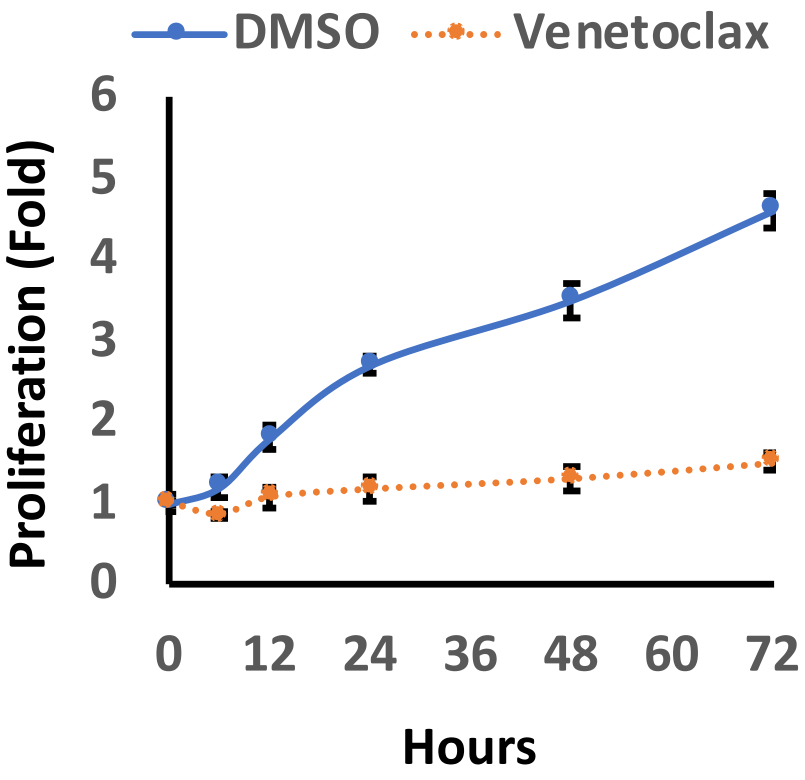

Supplementary Figure 7. Hepatic stellate cells (LX-2) are sensitive to Venetoclax. LX2 cell growth at the indicated times were measured using CelltiterGlo (n=3). The values at 0 hours were arbitrarily assigned as 1.

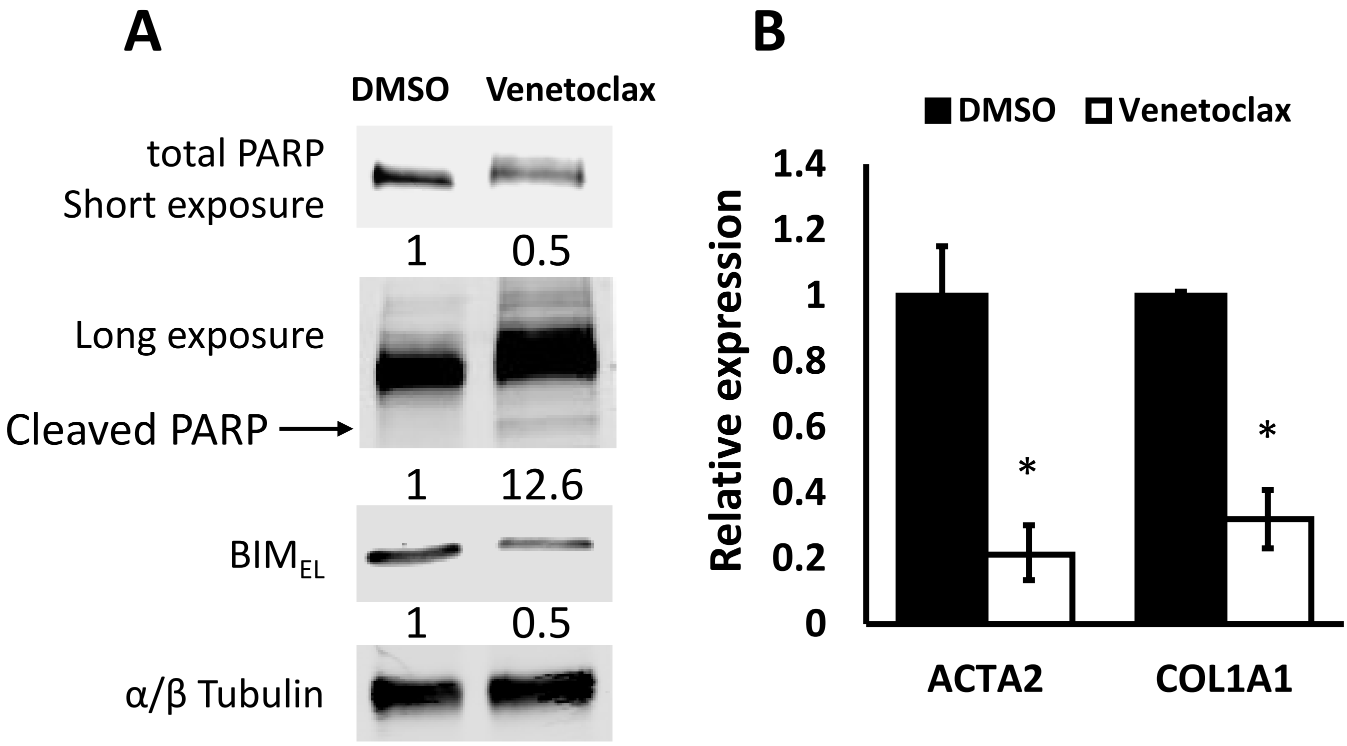

Supplementary Figure 8. Venetoclax promotes apoptosis in LX-2 cells. (A) Immunoblotting of the vehicle and Venetoclax (10μM) treated LX-2 cells. (B) The mRNA level of liver fibrosis markers (ACTA2 and COL1A1) were measured in the vehicle or Venetoclax treated LX-2 by RT-qPCR (n=3). *P<0.05, Student's t-test

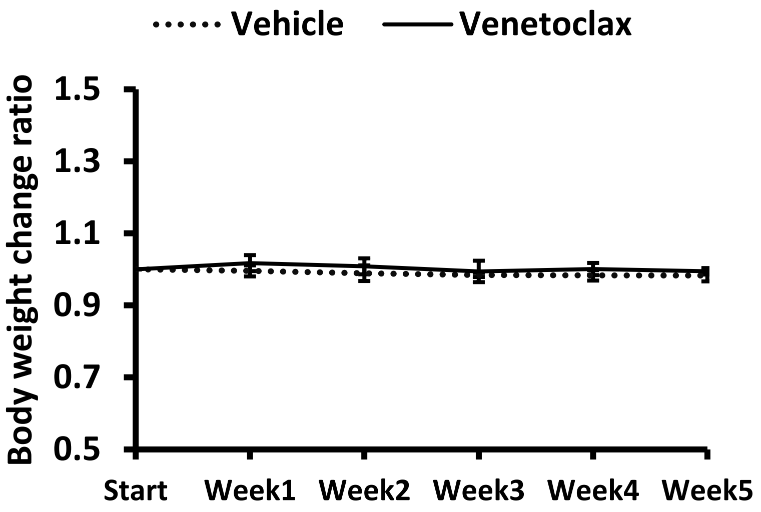

Supplementary Figure 9. Weekly changes in body weight of miR-122 KO mice treated with Venetoclax or vehicle. No significant body weight changes in body weight were observed up to 5 weeks of treatment (n=5 for each group). Mice also behaved normally during the treatment period.

Supplementary Table 1 Primer used for RT-qPCR and cloning

[1]**H & m ACTA2 RT-F**	5'- ATGCTCCCAGGGCTGTTTTC -3
**H & m ACTA2 RT-R**	5'- TCCCAGTTGGTGATGATGCC -3'
**H & m COL1A1 RT-F**	5'- GCAAGAATGGAGATGATGGGG -3'
**H & m COL1A1 RT-R**	5'- AAACCACTGAAGCCTCGGTGTC -3'
**ACTB RT-F**	5'- CTGGCACCACACCTTCTACAATG -3'
**ACTB RT-R**	5'- TAGCACAGCCTGGATAGCAACG -3'
**BCL2 RT-F**	5'- CGTCCTGCCTTCATTTATCCAG -3'
**BCL2 RT-R**	5'- TGTGATGTTTTCCCCTTCTCGG -3'
**c-MYC-RT-F**	5'- AACAACCGCAAGTGCTCCAG -3'
**c-MYC-RT-R**	5'- TCTCGTCGTTTCCTCAATAAGTCC-3'
**Hsa-BCL2 hnRNA-F**	5'- GCCCTGTGGATGACTGAGTA -3'
**Hsa-BCL2 hnRNA-R**	5'- CACTCCAACCCCCGCATCTC -3'
**Mmu-BCL2 RT-F**	5'- CGAGAAGAAGGGAGAATCACAGG -3'
**Mmu-BCL2 RT-R**	5'- ATGAATCGGGAGTTGGGGTCTG -3'
**Ctgf WT-F**	5'-CTAGCTAGCCGGGAGCGTATAAAAGCCAGC -3'
**Ctgf mut-R**	5'-GGGTCGGAGCTCCTCACGATCTGGCTGAGT-3'
**Ctgf mut-F**	5'-ACTCAGCCAGATCGTGAGGAGCTCCGACCC-3'
**Ctgf WT-R**	5'-CTAGCTAGCAGCGAGGAGCACCAAGGCGAG-3'

[1] H & m indicates primers were used in both human and mouse samples.

Supplementary Table 2. Antibody information

**BCL2**	Santa Cruz Biotech, #SC-7382
**BIM**	Cell signaling, #2819
**c-MYC**	Cell signaling, #13987
**PARP**	Cell signaling, #9532
**α/β Tubulin**	Cell signaling, #2148
**GAPDH**	Santa Cruz Biotech, #SC-365062
**α-SMA**	DAKO, #M085129-2

Supplementary Table 3. Commonly upregulated fibrotic genes between mouse and human

Symbol	P-value in Human	P-value in Mouse
MMP2	6.15E-08	1.02E-24
COL1A2	1.93E-07	6.93E-42
BCL2	2.83E-07	2.20E-04
COL4A2	1.01E-06	1.86E-30
COL4A1	3.62E-06	7.08E-24
COL1A1	8.46E-06	7.85E-47
PDGFA	2.69E-05	0.001
COL4A3	3.85E-05	0.0000187
COL5A1	6.39E-05	3.26E-20
COL3A1	6.99E-05	1.38E-39
COL6A3	1.99E-04	0.000911
TGFB2	2.07E-04	0.000201

Fibrotic genes that are commonly up-regulated in the human cirrhotic livers and miR-122 KO liver. Data were retrieved from Oncomine (https://www.oncomine.org/) and PubMed (GSE20610). Fibrotic genes were further filtered by Ingenuity® Pathway Analysis (IPA®).

Supplementary Table 4. Transcription factor binding motifs conserved in mouse and human at the BCL2 promoter region.

	**Binding motifs No.**	**Sequence**
Myc-Max	1	cgcggaaCACTTGattctgg
Max	1	ggaaCACTTGattc
E2F1	1	ggCGGCAGAt
SP1	2	ggGGGAGGgt; ggGGGAGGGt
MAZ	1	gGGGAGGG
AP2	1	caccccGCCTCCGGgc

Binding sequences were predicted by rVista 2.0 (https://rvista.dcode.org/).
